# Monitoring the efficacy and safety of three artemisinin based-combinations therapies in Senegal: results from two years surveillance

**DOI:** 10.1186/1471-2334-13-598

**Published:** 2013-12-20

**Authors:** Khadime Sylla, Annie Abiola, Roger Clément Kouly Tine, Babacar Faye, Doudou Sow, Jean Louis Ndiaye, Magatte Ndiaye, Aminata Colé LO, Kuaku Folly, Léon Amath Ndiaye, Oumar Gaye

**Affiliations:** 1 Département de Parasitologie-Mycologie, Faculté de Médecine, Université Cheikh Anta DIOP de Dakar, Dakar, Senegal

**Keywords:** Artemisinin combination therapy, Malaria, *Plasmodium falciparum*, Senegal

## Abstract

**Background:**

Malaria remains a major public health problem in developing countries. Then in these countries prompt access to effective antimalarial treatment such as Artemisinin based-Combination Therapies (ACT) proves to be an essential tool for controlling the disease. In Senegal, since 2006 a nationwide scaling up program of ACT is being implemented. In this context it has become relevant to monitor ACT efficacy and provide recommendations for the Senegalese national malaria control program.

**Methods:**

An open randomized trial was conducted during two malaria transmission seasons (2011 and 2012) to assess the efficacy and safety of three combinations: dihydro-artemisinin-piperaquine (DHAPQ), artemether-lumefantrine (AL) and artesunate-amodiaquine (ASAQ). The primary end point of the study was represented by a PCR adjusted adequate clinical and parasitological response (ACPR) at day 28. Secondary end points included: (i) a ACPR at days 35 and 42, (ii) a parasite and fever clearance time, (iii) ACTs safety and tolerability. The 2003 WHO’s protocol for antimalarial drug evaluation was used to assess each outcome.

**Results:**

Overall, 534 patients were randomized selected to receive, either ASAQ (n = 180), AL (n = 178) or DHAPQ (n = 176). The PCR adjusted ACPR at day 28 was 99.41% for the group ASAQ, while that was 100% in the AL and DHAPQ groups (p = 0.37). The therapeutic efficacy was evaluated at 99.37% in the ASAQ arm versus 100% in AL and DHAPQ arm at day 35 (p = 0.37). At day 42, the ACPR was 99.27% in the ASAQ group versus 100% for both AL and DHAPQ groups, (p = 0.36). No serious adverse event was noted during the study period. Also a similar safety profile was noted in the 3 study groups.

**Conclusion:**

In the context of scaling up of ACTs in Senegal, ASAQ, AL and DHAPQ are highly effective and safe antimalarial drugs. However, it’s remains important to continue to monitor their efficacy.

**Trial registration:**

PACTR 201305000552290.

## Background

Despite increasing efforts to control malaria, the disease is still a public health problem. According to the World Health Organization (WHO), there are 216 million new malaria infection cases per year in the world. Also, the disease causes 655 000 deaths per year in the world. More than 81% of the new infection cases and 90% of the deaths occur in Africa mainly in children under five years [1]. The emergence and spread of the *P. falciparum* resistance to the monotherapies such as chloroquine, sulfadoxine, pyrimethamine, has been a major obstacle for malaria control in sub-saharan countries. Then to deal with these resistance issues, the WHO recommended Artemisinin based-Combination Therapies (ACT) for the management of uncomplicated malaria cases [2]. ACTs reduce malaria related morbidity and mortality and the transmission of *Plasmodium falciparum* by acting on gametocytes and reducing the chances of development of drug resistance [3-5].

In Senegal, the National Malaria Control Program (NMCP) initiated in 2006 a nationwide scaling up program of ACT [5]. Several ACTs are currently being used in Senegal including Artemether-lumefantrine (AL) and Artesunate-Amodiaquine (ASAQ) as first line treatment and dihydro-artemisinin-piperaquine DHAPQ (Duocotexcin*) as second line treatment [6].

Recent studies demonstrated a decline in ACTs efficacy as well as artesunate monotherapy in the Asian region [7,8]. This raised to some concerns related to ACT efficacy in the context of scaling up antimalarial intervention in West African countries particularly in Senegal. Then it becomes relevant to monitor ACT efficacy in Senegal.

In Senegal, notification of adverse events has been a great challenge for the National Malaria Control Programme although a pharmacovigilance system for monitoring ACT drug related adverse events has been established for several years [9]. Thus there is a need to document the safety profile of commonly used ACTs in Senegal when scaling up these antimalarial drugs. This study was undertaken to assess the efficacy and safety of three artemisinin combinations therapies for the treatment of uncomplicated *Plasmodium falciparum* malaria in Senegal.

## Methods

### Study period and area

The study was carried out during two malaria transmission seasons in two health centers: (i) Deggo which is located 20 km form Dakar, the capital city and (ii) Keur Soce located 200 km of South from Dakar. In the areas around the health posts, malaria is highly seasonal during the rainy season (July to October) with a peak of transmission from September to December. *Plasmodium falciparum* is the predominant species and transmission is mainly due to *Anopheles gambiae s.l.* The enrollment of patients started in 2011 and was completed in 2012.

### Study design

The study was designed as an open randomized trial comparing three ACTs for the treatment of uncomplicated *Plasmodium falciparum* malaria: Artesunate-Amodiaquine (ASAQ), Artemether-Lumefantrine (AL) and Dihydroartemisinin-Piperaquine (DHAPQ). Randomization was permuted in blocks of 9. The primary endpoint was the PCR adjusted adequate clinical and parasitological response (ACPR) at day 28. Secondary end points included: (i) PCR adjusted ACPR at day-35 and day-42, (ii) the parasite clearance time, (iii) the fever clearance time and (iv) ACT tolerability and safety.

The study was conducted as part of a national surveillance program aimed at monitoring ACTs efficacy under routine conditions.

### Study population

Subjects were enrolled if their age was above 6 months and they presented with uncomplicated *Plasmodium falciparum* malaria with parasite density ranged from 1000 to 100,000 trophozoites/μl. Ability to take oral medication and written informed consent were required as part of the inclusion criteria. Patients presenting with mono-infection by another species or mixed infectation, severe vomiting, severe malnutrition, severe signs of malaria (such as severe anemia, convulsion, respiratory distress), a positive pregnancy test and patients who had a history of allergy to study drugs or did not given informed consent were excluded from the study.

### Antimalarial treatment

After inclusion, all patients were weighed and randomized to receive one of three study drugs for three days. The drugs were administered under the direct supervision of the medical staff. In case of vomiting within the 30 minutes following the first administration, the same dose was administrated again.

Participants who vomited a second time were excluded from the study and received intravenous quinine treatment in accordance with the national malaria control program guidelines (25 mg/kg/day for seven days 3 times daily). The dosages of the study drugs were as follows:

Artesunate-Amodiaquine (ASAQ): the tablet contains 4 mg/kg/day Artesunate (AS) plus 10 mg/kg/day Amodiaquine (AQ). The drug was given once a day. The dosage was adjusted according to the weight: one tablet per day containing 25 mg/67.5 mg (4.5 - 9 kg), one tablet per day containing 50 mg/135 mg (9 - 18 kg), one tablet per day containing 100 mg/270 mg (18 - 36 kg) and two tablets per day containing 100 mg/270 mg if weight was more than 36 kg.

Artemether-Lumefantrine (AL): the drug was not given with additional fat. The tablet contains 20 mg of Artemether plus 120 mg of Lumefantrine. The drug was given 2 times a day. The dosage was adjusted according to the weight: two tablets per day (5 – 14 kg); four tablets per day (15 – 24 kg); six tablets per day (25 – 45 kg) and eight tablets per day if weight was more than 45 kg.

Dihydroartemisinin-Piperaquine (DHAPQ): the tablet contains 40 mg of Dihydroartemisinin (DHA) plus 320 mg of Piperaquine (PQ). The drug was given once a day. The dosage was adjusted according to the age: 3 tablets per day if age was more than 16 years; 2 tablets per day if age was between 11 and 16 years and 1.5 tablets if the age was between 6 and 11 years.

### Data collection

After inclusion, patients were followed at day 0 (day of inclusion), 1, 2, 3, 7, 14, 21 and 28. A random sub-sample of study participants was followed up to day 35 and day 42 to assess the long term protective effect of each drug after curative doses. A clinical and biological assessment was performed for all patients. The 2003 WHO’s protocol for antimalarial drug efficacy evaluation was used [10].

#### ***Clinical assessment***

A clinical examination and an interview were performed before the inclusion. After inclusion and first dose administration, all patients were examined during the first 4 days. At each follow up visit, a clinical examination and interview to evaluate the patient’s clinical conditions as well as the occurrence of adverse events were done. Patients were seen by the medical team at any time if they did not feel well.

#### ***Biological assessment***

A blood sample was collected for thick and thin smears for all study patients. Both tests were used to determine the parasite density and the plasmodium species at the day 0, 1, 2, 3, 7, 14, 21, and 28. Both tests were repeated at day 35, 42 and other days of follow up to evaluate parasite clearance times.

To distinguish recrudescence form new infection, blood was collected on filter paper at day 0 and at day of parasite reappearance.

The haemoglobin level was determined at day 0 and day 7 using Sysmex XS 1000i automate. Creatinine, urea, bilirubin, aspartate amino-transferase (ASAT) and alanine-aminotransferase (ALAT) were also examined at day 0 and day 7 using automate A15 of Biosystems laboratories.

### Laboratory methods

#### ***Thick and thin smear***

Finger prick blood was used to collect blood samples. Thick and thin smears were stained with Giemsa. The parasite density was evaluated by counting the number of asexual parasites per 200 white blood cells and calculated per μl: number of parasites × 8000/200 assuming a white blood cell count of 8000 cells per μl. Thick and thin smears were negative after 100 field microscopics reading.

#### ***Haematological and biochemical assessment***

Haematological and biochemical parameters were performed at enrolment and day 7 to determine the haemoglobin level, the concentration of urea, creatinine, bilirubine, asparta-amino-transferase (AST) and alanine-amino-transferase (ALAT).

#### ***Polymerase chain reaction (PCR)***

The PCR was used to distinguish recrudescence from new infection in case of treatment failure. Nested PCR was conducted to compare the genetic polymorphism of *P falciparum* genes (Merozoïte Surface Protein): *MSP1* and *MSP2*[11]. Recrudescence was defined as at least one identical allele for each of the two markers in the pre-treatment and post-treatment samples. New infections were diagnosed when all alleles for at least one of the markers differed between the two samples.

### Definition of early and late parasitological failure

#### ***Early treatment failure***

This was defined as a development of danger signs or severe malaria on days 1–3 in the presence of parasitaemia, a patient with parasitaemia on day 2 higher than the day 0 count irrespective of axillary temperature; parasitaemia on day 3 with axillary temperature ≥ 37.5°C and parasitaemia on day 3 that is ≥ 25% of count on day 0.

#### ***Late parasitological failure***

This was defined as a presence of parasitaemia on any day from day 7 to day 28 and axillary temperature < 37.5°C, without previously meeting any of the criteria of early treatment failure or late clinical failure.

#### ***Adequate clinical and parasitological response (ACPR)***

The ACPR was defined as an absence of parasitaemia on day 28 irrespective of axillary temperature without previously meeting any of the criteria of early treatment failure, late clinical failure or late parasitological failure [2].

### Statistical methods

Based on an expected therapeutic effect of AL not low than 95% [12] assuming a non-inferiority margin of 7% (two side) and power at 80%, using a 95% confidence level and accounting for 10% of lost to follow up, sample size for each study arm was evaluated at 155 participants.

Data collected were entered into Excel software and the analysis was done with Stata IC 12 software. Intention to treat and per protocol analysis were performed.

The intention to treat included all randomized subjects who took at last one full dose and had one post-baseline efficacy without major protocol deviation. The per protocol analysis include all subjects who received the three dose and had no major protocol deviation.

Data were analysed by estimation of difference in proportion according to a 95% confidence interval. Groups were compared using Chi Square test or Fisher exact test for categorical variables and Student’s t-test for continuous variables when these tests were applicable. Otherwise, non- parametric tests (Mann–Whitney, Kruskall-Wallis) were used.

The cumulative incidence of failure rate was calculated in each group and compared using Kaplan Meier method. Changes in biological parameters from day 0 to day 7 were calculated and compared between treatment arms using Bonferroni test.

Statistical significance for all tests was set at 0.05 (*p value < 0.05 two side)*.

### Ethical considerations

This study was conducted according to the Declaration of Helsinki and existing national legal and regulatory requirements. The protocol was reviewed and approved by the Senegalese National Ethical Committee (*Conseil National d’Ethique et de Recherche en Santé).* Written informed consent was obtained from each participant or their parent or guardian for particiapants with an age below 18 years. The study was registered at the Pan African Clinical Trial Registry: registration number: PACTR201305000552290.

## Results

### Trial profile

**Figure 1:**
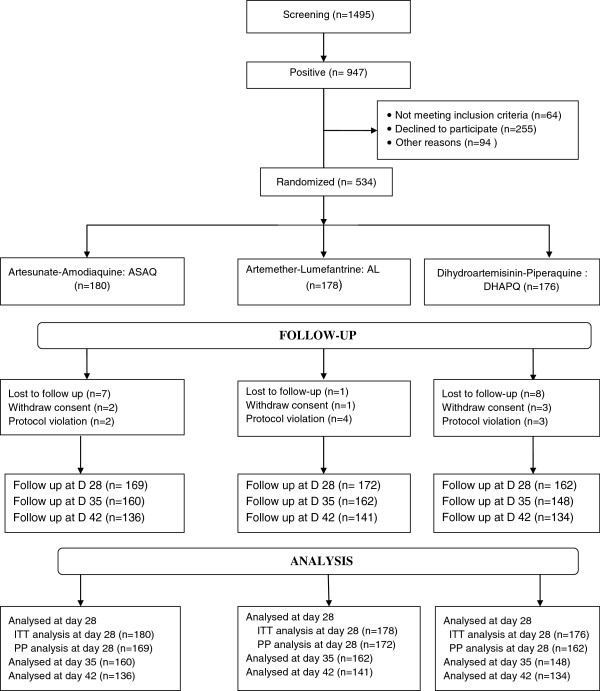
Trial profile.

Overall, 1495 febrile patients were screened for malaria. 947 of them were positive for *Plasmodium falciparum* malaria. For positives subjects, 534 of them meet the inclusion criteria: 180 in the ASAQ arm, 178 in the AL arm and 176 in the DHAPQ arm. Withdrawal of consent was noted in 2 patients in the ASAQ arm, 1 patient in the AL arm and 3 patients in the DHAPQ arm. During the follow up, 7 patients in ASAQ group, 1 patient in AL group and 8 patients in DHAPQ group were lost to follow up. Protocol violations were observed in 2 subjects in ASAQ group, 4 patients in AL group and 3 subjects in DHAPQ group. At the end of the study, 534 patients were included in ITT analysis and 503 patients in PP analysis at day 28. The PP analysis at day 35 and day 42 concerned respectively 470 and 411 patients (Figure 1).

### Baseline characteristics of subjects at inclusion in the three treatment groups

At inclusion, the three groups were comparable in term of age, weight, sex ratio, temperature and parasitemia. The median age was 14 years in the ASAQ and DHAPQ groups and 13 years in the AL group. The sex ratio was 1.85, 1.17 and 1.41 respectively in the ASAQ, AL and DHAPQ arm. The mean weight in each group was 38.9 ± 18 kg, 37.5 ± 20 kg and 42.4 ± 19 kg for ASAQ, AL and DHAPQ, significantly higher in the last group. The mean temperature was almost similar in the three treatment groups. The proportion of subjects with fever on admission was 75% (135/180), 75.8% (135/178) and 76.14% (134/176) respectively in ASAQ, AL and DHAPQ group.

The median parasitemia was 13132.5 trophozoites/μl in ASAQ arm, 26192.5 trophozoites/μl in AL arm and 21347.5 trophozoites/μl in DHAPQ arm (Table 1).

**Table 1:** Baseline characteristics of subjects at the inclusion in the three treatment groups

**Parameters**	**DHAPQ (N = 176)**	**AL (N = 178)**	**ASAQ (N = 180)**
**Age (median ± SD, years)**	14 ± 10	13 ± 12	14 ± 12
**Sex ratio (F/M)**	1.41	1.17	1.85
**Weight (mean ± SD, Kg)**	42.4 ± 19	37.5 ± 20	38.9 ± 18
**Temperature (mean ± SD)**	38.1 ± 1.1	38 ± 1.08	38 ± 2
**Patients with fever**	76.14%	75.8%	75%
**Parasitemia (median ± trophozoites/μL)**	21347.5	26192.5	13132.5
**Hb mean (g/dl)**	11.88	11.89	11.48
**Anemia (Hb < 11 g/dl) (%)**	24.43	22.47	29.44
**ALAT (UI/L, mean ± SD)**	19	21	20.76
**ASAT (UI/L, mean ± SD)**	34.37	33.52	32.17
**Patients with ALAT < 40 UI/L (%)**	89.77	88.20	84.44
**Patients with ASAT < 40 UI/L (%)**	80.68	83.15	88.20
**Mean bilirubin**	1.3	1.5	0.81
**Patients with normal level of bilirubin (0.23 – 1 mg/dl)**	77.27	79.78	79.44
**Mean creatinine (mg/l)**	8.15	8.75	7.9
**Patients with normal level of creatinine (6 – 14 mg/l) (%)**	77.27	80.34	93.33

### Biological characteristics of patients at inclusion

No significant difference was observed in the mean level of haemoglobin, creatinine, bilirubin and liver enzymes such as ASAT and ALAT in the three groups treatment (Table 1).

### Therapeutic efficacy

There were no early treatment failures and cure rates, both PCR uncorrected and corrected, for all three treatment groups were higher than 95% by ITT and PP analysis, with no significant differences observed between the groups (Table 2).

**Table 2:** Treatment outcomes of ASAQ, AL and DHAPQ at day 28

**Outcome**	**Uncorrected**				**PCR-Corrected**			
	**DHAPQ (N = 176)**	**AL (N = 178)**	**ASAQ (N = 180)**	**p-value**	**DHAPQ (N = 176)**	**AL (N = 178)**	**ASAQ (N = 180)**	**p-value**
	**% (95% CI)**	**% (95% CI)**	**% (95% CI)**		**% (95% CI)**	**% (95% CI)**	**% (95% CI)**	
**Intention to treat analysis**								
***Early treatment failure***	00	00	00		00	00	00	
***NA***	6/176 (3.41%)	2/178 (1.12%)	4/180 (2.22%)		6/176 (3.41%)	2/178 (1.12%)	4/180 (2.22%)	
***Late parasitological failure***	1/176 (0.57%)	1/178 (0.56%)	2/180 (1.1%)		00	00	1/180 (0.56%)	
***ACPR***	169/176 (96.02%)	175/178 (98.31%)	174/180 (96.67%)	0.43	170/176 (96.59%)	176/178 (98.88%)	175/180 (97.22%)	0.35
	[89.3-99.9]	[87.3-99.9]	[88.8-99.9]		[88.7-99.9]	[86.8-99.9]	[88.4-99.9]	
**Per protocol analysis**	**DHAPQ (N = 162)**	**AL (N = 172)**	**ASAQ (N = 169)**		**DHAPQ (N = 162)**	**AL (N = 172)**	**ASAQ (N = 169)**	
	**% (95% CI)**	**% (95% CI)**	**% (95% CI)**		**% (95% CI)**	**% (95% CI)**	**% (95% CI)**	
***Early treatment failure***	00	00	00		00	00	00	
***Late parasitological failure***	1/162 (0.62%)	1/172 (0.58%)	1/169 (0.59%)		00	00	1/169 (0.59%)	
***ACPR***	161/162 (99.38%)	171/172 (99.42%)	168/169 (99.41)	0.99	162/162 (100%)	172/172 (100%)	168/169 (99.41)	0.37
	[85.7-99.9]	[86.1-99.9]	[86–99.9]		[85.2-99.9]	[85.6-99.9]	[86–99.9]	

NA: Not Applicable, ACPR: Adequate Clinical and Parasitological Response.

**Figure 2:**
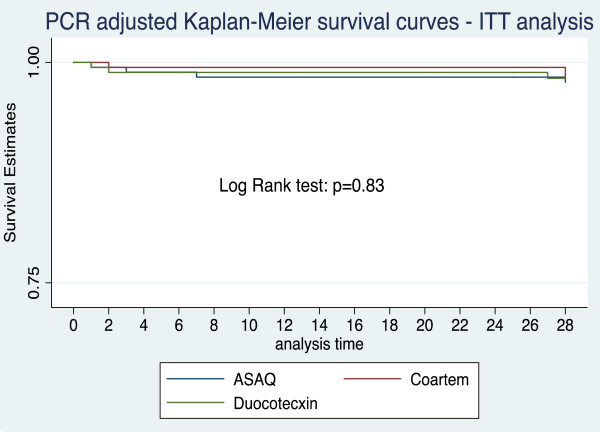
Kaplan Meier survival estimates of efficacy in three treatment groups in ITT analysis at day 28.

The Kaplan Meier survival analysis resulted in a very similar cumulative incidence failure rate at day 28 in all three groups (log rank test, p = 0.83) (Figure 2).

A very low rate of late parasitological failures were detected (Table 2).

There were 88% (n = 470) and 77% (n = 411) of all patients seen at day 35 and day 42 respectively. Again very good cure rates were observed with no significant difference detected between the groups (Table 3).

**Table 3:** Treatment outcomes of ASAQ, AL and DHAPQ at day 35 and day 42

**Outcomes**	**Uncorrected**				**PCR-corrected**			
	**DHAPQ (N = 148)**	**AL (N = 162)**	**ASAQ (N = 160)**	**p value**	**DHAPQ (N = 148)**	**AL (N = 162)**	**ASAQ (N = 160)**	**p value**
	**% (95% CI)**	**% (95% CI)**	**% (95% CI)**		**% (95% CI)**	**% (95% CI)**	**% (95% CI)**	
**Day 35**								
***Late parasitological failure***	1/148	1/162	2/160		00	00	1/160 (0.625%)	
	(0.67%)	(0.62%)	(1.25%)					
***ACPR***	147/148	161/162	158/160	0.79	148/148	162/162	159/160	0.37
	(99.33%)	(99.38%)	(98.75%)		(100%)	(100%)	(99.375%)	
	[85.1-99.9]	[85.7-99.9]	[86.2-99.9]		[84.5-99.9]	[85.2-99.9]	[85.6-99.9]	
**Day 42**	**DHAPQ (N = 134)**	**AL (N = 141)**	**ASAQ (N = 136)**		**DHAPQ (N = 134)**	**AL (N = 141)**	**ASAQ (N = 136)**	
	**% (95% CI)**	**% (95% CI)**	**% (95% CI)**		**% (95% CI)**	**% (95% CI)**	**% (95% CI)**	
***Late parasitological failure***	1/134	4/141	4/136		00	00	1/136	
	(0.75%)	(2.84%)	(3%)				(0.73%)	
***ACPR***	133/134	137/141	132/136	0.38	134/134	141/141	135/136	0.36
	(99.25%)	(97.16%)	(97%)		(100%)	(100%)	(99.27)	
	[84.4-99.9]	[86.6-99.9]	[87.1-99.9]		[83.7-99.9]	[84.2-99.9]	[84.5-99.9]	

ACPR, Adequate Clinical and Parasitological Response; NA, Not Applicable.

### Fever and parasite clearance

Fever clearance was similar in the three treatment groups. At inclusion, the proportion of subjects with fever was 75% (135/180), 75.8% (135/178) and 76.14% (134/176) respectively in ASAQ, AL and DHAPQ group. The difference was not significative at inclusion between the groups (p = 0.96). After first dose administration, 3.8% (7/180) patients in ASAQ group and 3.4% (6/176) patients in DHAPQ group were found with fever. The proportion of patients with fever was more important in the AL group 8.4% (15/178) with no significant difference (p = 0.06). The fever clearance was noted at day 2 after the first dose treatment.

The three treatments showed rapid parasite clearance time. The parasitemia at inclusion was 13132.5 trophozoites/μl in ASAQ group, 26192.5 trophozoites/μl in AL group and 21347.5 trophozoites/μl in DHAPQ group. At day 1 after first dose administration, the parasitemia decreased to 1139.8 trophozoites/μl in ASAQ group, 583.03 trophozoites/μl in AL group and 277.73 trophozoites/μl in DHAPQ group. The proportion of patients remaining parasitemic at day 1 was 75% (135/180), 66.8% (119/178) and 48.8% (86/176) respectively in ASAQ, AL and DHAPQ group (p < 10^-3^). At day 2, this proportion was 3.3% (6/180), 6.7% (12/178) and 2.3% (4/176). The difference was not significative at day 2 after first dose administration (p = 0.08) between the three treatments groups. Complete parasite clearance was obtained at day 3 after inclusion.

### Clinical and biological tolerance

The three treatments were well tolerated during the study period. No serious adverse event was observed. No patient died during the study period and no signs of neurotoxicity were observed. The main adverse events were minor and they were represented by vomiting, abdominal pain, herpes labialis, dizziness and diarrhea.

Abdominal pains were more frequent in ASAQ group 16.66% (30/180) versus 10.79% (19/176) in DHAPQ group and 7.86% (14/178) in AL group (p = 0.31). Vomiting was more frequent in DHAPQ group 5.68% (10/176) compared to AL group 2.45% (4/178) and ASAQ group 1.66% (3/180) (p = 0.06). Labial herpes was more frequent in AL group 3.37% (6/178).

Biological tolerance was good during the three treatments. No major biological disorder was observed.

The mean of haemoglobin was lower at day 7 in the three treatments groups compared to hemoglobin mean at day 0. The difference between the three treatment groups at day 7 was not significative (p = 0.19). Anemia was most frequent at day 7 in three groups compared to inclusion. This was more important in AL group (70.79%). The difference was not significative between the three groups (p = 0.31).

An improvement of liver function was observed between day 0 and day 7. The number of patients with normal transaminase was higher at day 7 compared to the enrollment.

Regarding the mean of creatinine, no significant variation was observed between day 0 and day 7 in the three treatment arms. A significant decrease of bilirubin level was noted at day 7 in the three treatment arms (Table 4).

**Table 4:** Patients’ biological profile at day 7 in the three treatment groups

**Parameters**	**DHAPQ (N = 176)**	**AL (N = 178)**	**ASAQ (N = 180)**	**p value**
**Hb mean (g/dl)**	10.74	10.75	10.96	0.19
**Anemia (Hb < 11 g/dl) (%)**	63.64	70.79	69.44	0.31
**ALAT (UI/L, mean ± SD)**	16.42	17.34	18.19	0.58
**ASAT (UI/L, mean ± SD)**	25.6	23.9	25.8	0.09
**Patients with ALAT < 40 UI/L (%)**	90.34	93.82	91.67	0.47
**Patients with ASAT < 40 UI/L (%)**	90.91	95.51	98.89	0.002
**Mean bilirubin (mg/dl)**	0.56	0.58	0.64	0.02
**Patients with normal level of bilirubin (0.23 – 1 mg/dl) (%)**	96.59	96.07	93.89	0.42
**Mean creatinine (mg/l)**	7.74	7.31	7.72	0.93
**Patients with normal level of creatinine (6 – 14 mg/l) (%)**	96.59	96.07	100	0.03

At the pair analysis, the haemoglobin mean decreased from day 0 to day 7 with no statistical difference between AL and DHAPQ treatment groups (p = 1). From day 0 to day 7, this mean decreased to 1.1 g/dl versus 0.52 g/dl respectively in AL and ASAQ groups. Haemoglobin level decreased to 1.4 g/dl from day 0 to day 7 in the DHAPQ group versus 0.52 g/dl in the ASAQ group (p < 0.001). Overall the ALAT mean decreased from day 0 to day 7 in the three treatments groups. No statistical difference was noted between ASAQ (2.57 UI/L) and AL (3.65 UI/L) groups (p = 1) and between AL (16.2 UI/L) and DHAPQ (2.58 UI/L) groups (p = 1). From day 0 to day 7, the mean ALAT decreased to 3.65 UI/L in AL group versus 2.58 UI/L in DHAPQ group (p = 0.48). The mean ASAT decreased to 6.4 UI/L in ASAQ group versus 9.5 UI/L in AL group from day 0 to day 7 (p = 0.54). Same tendency was noted in DHAPQ (8.7 UI/L) and AL (9.5 UI/L) groups from day 0 to day 7 (p = 1). There was no significant difference in ASAT level from day 0 to day 7 in ASAQ and DHAPQ arms (p = 0.94). From day 0 to day 7, the mean production of creatinin decreased to 0.18 mg/l in ASAQ group versus 0.41 mg/l in DHAPQ group (p = 1). In AL group this mean decreased to 1.39 mg/l from day 0 to day 7 versus 0.18 mg/l in ASAQ group (p = 0.77). Regarding AL group versus DHAPQ group, the mean creatinin decreased respectively to 1.39 mg/L and 0.41 mg/l from day 0 to day 7 (p = 1).

Overall, the mean bilirubin decreased from day 0 to day respectively to 0.17 mg/dl, 0.91 mg/dl and 0.78 mg/dl in ASAQ, AL and DHAPQ group. The difference was significative between ASAQ and AL groups and between ASAQ and DHAPQ groups (p < 10^-3^). There was not significative between AL and DHAPQ groups (p = 0.46) (Table 5).

**Table 5:** Biological parameter changes from day 0 to day 7 by treatment arms

**Treatment Group**	**Point estimate changes of biological parameters from day 0 to day 7 (95% IC)**	**Mean difference**	**p value**
**Haemoglobin (g/dl)**			
**ASAQ**	0.52 ± 0.11 (0.28 – 0.75)	ASAQ ≠ AL: 0.62	<10^-3^
**AL**	1.1 ± 0.09 (0.96 – 1.32)	ASAQ ≠ DHAPQ: 0.62	<10^-3^
**DHAPQ**	1.1 ± 0.08 (0.98 – 1.31)	AL ≠ DHAPQ: 0.001	1
**ALAT (UI/L)**			
**ASAQ**	2.57 ± 1.86 (-1.1 – 6.2)	ASAQ ≠ AL: 1.08	1
**AL**	3.65 ± 1.2 (1.2 – 6.1)	ASAQ ≠ DHAPQ: 0.008	1
**DHAPQ**	2.58 ± 1.5 (-0.3 – 5.5)	AL ≠ DHAPQ: -1.07	1
**ASAT (UI/L)**			
**ASAQ**	6.37 ± 0.4 (5.4 – 7.3)	ASAQ ≠ AL: 3.17	0.54
**AL**	9.55 ± 1.8 (5.9 – 13.1)	ASAQ ≠ DHAPQ: 2.38	0.94
**DHAPQ**	8.75 ± 2.2 (4.3 – 13.2)	AL ≠ DHAPQ: -0.79	1
**Creatinin (6–14 mg/l)**			
**ASAQ**	0.18 ± 0.7 (-1.2 – 1.6)	ASAQ ≠ AL: 1.21	0.77
**AL**	1.39 ± 0.5 (0.3 – 2.4)	ASAQ ≠ DHAPQ: 0.22	1
**DHAPQ**	0.41 ± 0.9 (-1.4 – 2.3)	AL ≠ DHAPQ: -0.98	1
**Bilirubin (0.23 - 1 mg/dl)**			
**ASAQ**	0.17 ± 0.03 (0.1 – 0.23)	ASAQ ≠ AL: 0.74	<10^-3^
**AL**	0.91 ± 0.07 (0.7 – 1.06)	ASAQ ≠ DHAPQ: 0.61	<10^-3^
**DHAPQ**	0.78 ± 0.07 (0.6 – 0.9)	AL ≠ DHAPQ: -0.12	0.46

Overall, the pair analysis showed an improvement of liver and kidney function from day 0 and day 7.

## Discussion

This study was undertaken to assess the efficacy and the safety of the three artemisinin combination therapies commonly used in Senegal. The study showed that AL, DHAPQ and ASAQ are highly effective for the treatment of uncomplicated *Plasmodium falciparum* malaria. These findings are consistent with results reported from other trials. In Senegal, *Tine et al.* in 2010 reported a cure rate of 96.7% at day 28 for AL in a clinical trial assessing the efficacy and tolerability of new formulation of Artesunate-Mefloquine [13]. *Faye et al.* obtained a cure rate of 97% for AL in a multicentric study (Senegal and Ivory Coast) from September 2007 to November 2008 [14].

*Menan et al.* from, in a multicentric study including Cameroon, Ivory Coast and Senegal September 2008 to February 2009, obtained a PCR adjusted ACPR at day 28 of 99% in AL group [15]. *Makanga et al.* obtained a therapeutic efficacy for AL above 95% for the treatment of uncomplicated malaria [16]. Regarding ASAQ combination, *Faye et al.* in 2008 in a multicentric study (Senegal, Cameroon and Ivory Coast) evaluating the non-inferiority of the new paediatric formulation of Artesunate/Amodiaquine versus Artemether/Lumefantrine for malaria treatment reported a therapeutic efficacy at day 28 at 98.7% when ASAQ was given to children under 5 years of age [17].

*Ndiaye et al.*, in randomized trial assessing the efficacy of a fixed dose of ASAQ combination from March to December 2006, showed an adjusted ACPR at day 28 more than 98% [18].

*NDounga et al.* in 2005 in Brazaville, obtained 94.4% of cure rate at day 28 when ASAQ combination was given to children [19]. *Zwang et al.* in a multi-centre analysis of the efficacy of ASAQ combination showed good ACPR after PCR correction. For DHAPQ combination similar results were obtained [20]. *Yavo et al.* from November 2006 to May 2008 in multi-centre study assessing the efficacy of DHAPQ obtained 99.5% of ACPR after PCR correction [21].

In Thaïland, *Ashley et al.* between July 2002 and April 2003 showed a cure rate of 98.3% in the DHAPQ group [22]. Many studies have reported good efficacy of DHAPQ combination for malaria treatment [23-25]. The three antimalarial drugs remained highly effective at days 35 and 42. Compared to others drugs, DHAPQ has good cure rate more than 98%. This result was demonstrated by *Grande et al.* and by *Zwang et al*[26,27].

After the administration of the three ACT used in this study, it resulted in a rapid decrease of fever and parasite. The fever and parasite clearance was obtained respectively at day 2 and day 3 after the initial dose. Similar results were obtained by *Tine et al.*, *Faye et al.* in Senegal and *Gbotosho et al.* in Nigeria [13,14,28].

The three antimalarial treatments were well tolerated with a similar safety profile. Indeed, no serious adverse event was noted. The main adverse events were minor and they were represented by vomiting, abdominal pain, herpes labialis, dizziness and diarrhea. However abdominal pains were more frequent in patients treated with ASAQ, vomiting was more frequent in patients treated with DHAPQ and labial herpes was more frequent in AL group. Previous study demonstrated that these ACTs are well tolerated [13-15].

No major biological disorder was observed in our study. Anemia was most frequent at day 7 in three groups compared at day 0. Similar results were noted by others study. *Olliaro et al.* observed a decrease of haemoglobin level at day 7 after malaria treatment [29]. Same trends were noted by *Price et al.* and by *Zwang et al.*[30,31].

In Senegal, notification of adverse events has been a great challenge for the National Malaria Control Programme although a pharmacovigilance system to monitor ACT drug related adverse events has been established since 2006 [9]. This study provided scientific evidence that can contribute to supplement existing data regarding on ACT safety in Senegal.

### Study limitation

AL was not given with fat diet in our study. This could explain the decrease of the efficacy. However many studies have reported good efficacy when AL was given with fat. Thus Ashley et al. in pharmacokinetic study of AL in 2002 showed that the high-fat allowed having a good absorption of the drug. This had resulted in an increase of therapeutic efficacy [32].

*Mayxay M et al.*, in the efficacy study of AL in Southern Laos between June and November from 2008–2010 showed good efficacy of AL with a cure rate more than 95% when AL was given with fatty food [33].

Many studies reported an increase of haemoglobin level from day 7 to 28 after treatment with ACT [20,31]. In this study haemoglobin level was assessed at day 0 and day 7. Additional haemoglobin dosages at day 14, 21 and 28 would provide more information on haemoglobine changes after treatment with ACTs; theses assessments were not done in the study.

## Conclusions

DHAPQ, AL and ASAQ are highly effective and safe antimalarial drugs. These ACTs remain useful antimalarial interventions for effective malaria control. It is however important to continue to monitor their efficacy in the context of scaling up of ACTs in Senegal.

## Competing interests

The authors declare that they have no competing interest.

## Authors’ contributions

KS, RCT, BF, DS, JLN, OG conceived and designed the study. KS and MN monitored the data collection. KF and LAN collected data in the site. KS and RCT analysed the data. MN, ACL, AA were responsible for the PCR analysis. KS and AA wrote the first draft of the manuscript. All authors read and approved the final manuscript.

## Pre-publication history

The pre-publication history for this paper can be accessed here:

http://www.biomedcentral.com/1471-2334/13/598/prepub
